# Bipolar disorder, a precursor of Parkinson's disease?

**DOI:** 10.1590/s1980-5764-2016dn1004018

**Published:** 2016

**Authors:** Tânia M.S. Novaretti, Nathália Novaretti, Vitor Tumas

**Affiliations:** 1 MD PhD. Clínica de Urologia - UROMED, Marília, SP, Brazil; 2 MD. Department of Neuroscience and Behavior, Faculty of Medicine of Ribeirão Preto, USP, SP, Brazil.; 3 MD PhD. Department of Neuroscience and Behavior, Faculty of Medicine of Ribeirão Preto, USP, SP, Brazil.

**Keywords:** Parkinson's disease, bipolar disorder, serotoninergic pathway, dopamine

## Abstract

Parkinson's disease is a neurodegenerative disorder predominantly resulting from dopamine depletion in the substantia nigra pars compacta. Some psychiatric disorders may have dopaminergic dysfunction as their substrate. We describe a well-documented case of Parkinson's disease associated with Bipolar Disorder. Although there is some knowledge about the association between these diseases, little is known about its pathophysiology and correlation. We believe that among various hypotheses, many neurotransmitters are linked to this pathophysiology.

## Abstract (pt)

A doença de Parkinson é um distúrbio neurodegenerativo resultante predominantemente da depleção de dopamina na substância negra pars compacta. Alguns transtornos psiquiátricos podem ter como substrato a disfunção dopaminérgica. Nós descrevemos um caso bem documentado de doença de Parkinson associado a Transtorno Bipolar. Embora haja algum conhecimento sobre a associação entre essas doenças, pouco se sabe sobre sua fisiopatologia e correlação. Acreditamos que dentre várias hipóteses, muitos neurotransmissores estão ligados a esta fisiopatologia.

## INTRODUCTION

Parkinson's disease (PD) is a neurodegenerative disorder characterized by motor findings such as resting tremor, bradykinesia and rigidity. Non-motor features of PD have received greater attention in the past two decades owing to their recognized contribution to disability.^1^ Wider biological changes occur in PD and include increased oxidative and nitrosative stress, immune-inflammatory processes, tryptophan catabolites and alterations in serotoninergic and melatoninergic pathways.^2^ Mood disturbance, and especially major depressive disorder, is a common non-motor condition in PD, with an average prevalence of 25-40% in outpatient settings.^3^ Bipolar disorder (BD) is a psychiatric disorder characterized by recurrent episodes of mania/hypomania and depression.^4^ Recently, some studies have demonstrated the association between BD and PD; however, although there is some knowledge about risk factors associated with BD in PD (5), little is known about its pathophysiology,the mechanisms involved in BD or whether it can be considered a non-motor symptom or comorbidity of PD

## METHODS

Case report and literature revision.

## CASE REPORT

**Figure 1:**
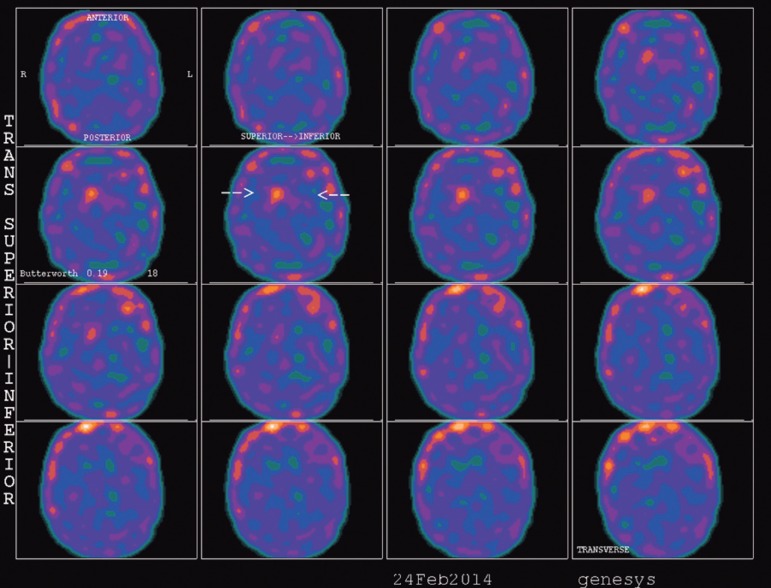
Single-Photon Emission Computed Tomography (SPECT) of patient using the radiotracer TRODAT showing right striatum of 0.84 (0.64-1.0) and left striatum <0.20(0.64-1.0), suggestive of Parkinson's disease.

We describe a case of PD in the context of BD: GJV, a 43-year-old male, was referred to our service in 2014 with a history of depression since 2003 and maniac episode after antidepressant treatment without mood stabilizer. After this episode, although adequately treated with mood stabilizing drugs including lithium (for a few months, subsequently withdrawn after diarrhea), anticonvulsants (valproate and carbamazepine) and atypical antipsychotics (risperidone and quetiapine), he never returned to a euthymic state. On his first visit, while in use of venlafaxine 300 mg and quetiapine 300 mg, he reported depression and had noticed tremor in his right hand the previous year. He had right persistent, involuntary resting tremor, bradykinesia and rigidity. Single-Photon Emission Computed Tomography (SPECT) using the radiotracer TRODAT disclosed a right striatum of 0.84 (0.64-1.0) and left striatum <0.20(0.64-1.0) compatible with PD (Figure 1). After PD treatment with levodopa medication 500 mg/day and pramipexole 2 mg/day, the psychiatric disease was stabilized using venlafaxine 75 mg/day and lamotrigine 100 mg/day. We believe that the PD treatment helped promote mood stabilization

## DISCUSSION

Parkinson's disease is a neuropsychiatric disorder characterized by both motor and non-motor symptoms. Depression is probably the most common non-motor symptom in PD.^3^ DSM-IV-defined major depressive disorder occurs in 17% of PD patients and minor depression in 22%.^6,7^ Cannas et al. (2002) described five patients with PD and BD, but their patients developed the psychiatric disorder a few years after starting dopaminergic therapy in the presence of a mild motor disability and a mild cognitive impairment. BD was diagnosed retrospectively by examining all available clinical data, the typical history and DSM-IV criteria.^8^ Although one of the manifestations of bipolar disorder is depression, a 2010 study in Brazil found a prevalence of Bipolar I Disorder of 1% and of Bipolar II Disorder of 5% in patients with PD, with no difference to the rate of bipolar disorders found in the general population.^9^ In recent years, there has been increased reporting of cases of BD and PD, but still without an exact correlation of cause or consequence. Several mechanisms are involved in BD and PD neurodegeneration, such as inflammatory process, cytokines, epigenetic alterations and neurotransmission dysfunction, which will be the focus of our discussion. Dopamine, PD and BD: the dopamine (DA) system has been demonstrated to be a particularly age-sensitive neurotransmitter network. During the course of normal aging, the number of DA neurons, receptors, and transporters declines.^10-14^ As the DA system has a central role in higher-order cognitive functions, a correlative triad among aging, DA integrity, and cognition may be proposed.^15^ Several lines of evidence implicate the dopaminergic system in the pathogenesis of both maniac and depressive episodes and increased dopaminergic function has been consistently found after long-term treatment with antidepressants.^16^ In a 2005 study, Leszczynska-Rodziewicz et al. found no significant association between the polymorphisms of dopamine receptors, type D2, and bipolar affective disorder in a Polish population.^17^ Dopamine has been implicated in the neurobiology of BD,^18^ however, we must consider that a single monoamine is responsible for the heterogeneous phenotypes of this neuropsychiatric disorder. For example, there is no decreased frequency of psychosis in PD and, even though dopamine plays a relevant role in schizophrenia, psychosis is not necessarily related to dopamine replacement therapy.^19^ BD, and particularly dementia in late-life bipolar disorder, was initially considered as comorbid Alzheimer's disease (AD).^20^ In 2016, Forlenza et al. investigated whether cognitively impaired older adults with BD might have a profile of CSF biomarkers similar to that reported in dementia and in MCI due to AD, i.e., lower concentrations of Aþ1-42 and higher concentrations of T-tau and P-Tau.^21^ Their analysis of CSF failed to support the supposition of a common biological signature associated with cognitive deterioration in BD and AD. The phenotypic endpoint of PD is classically associated with loss of dopaminergic neurons in the substantia nigra, with many susceptibility genes and environmental factors being associated with PD.^22^ Dopaminergic degeneration is a hallmark of PD, which causes various symptoms affected by corticostriatal circuits. Although dopaminergic degeneration is the most important pathologic marker of PD, it is followed by various types of functional progressive degeneration in the whole brain involving the limbic system and neocortex.^23^ Several studies have reported that non-motor symptoms such as cognitive dysfunction in PD were related to widespread cortical atrophy in frontoparietal, limbic and cerebellum areas.^24-26^ Anterior striatal dopaminergic denervation is related to non-motor symptoms including sensory, neuropsychiatric and cognitive functions.^27-29^ Glutamate and BD: BD has been consistently associated with glutamatergic system abnormalities.^30,31^ Pharmacological studies reinforce the association between BD and the glutamatergic system by reporting that first line agents to treat BD, such as lithium, valproate, carbamazepine, and lamotrigine, also modulate the glutamatergic system.^32^ Soeiro-de-Souza et al., 2013, evaluated glutamate levels in the Anterior Cingulate Cortex (ACC) and found that BD subjects during euthymia had higher glutamate levels compared with healthy controls.^33^ ACC shares extensive anatomical connections with the amygdala; subiculum; hypothalamus; accumbens; ventral tegmental area; substantia nigra; raphe; locus coeruleus; periaqueductal gray; and brainstem autonomic nuclei, and other areas of the orbitomedial pre-frontal cortex.^34,35^ These structures are implicated in the modulation of emotional behavior, raising the possibility that abnormal synaptic interactions between these areas and the ACC may contribute to disturbances in emotional processing or regulation.^36^ Serotonin, PD and BD: the serotonergic hypothesis is one of the few hypotheses attempting to link the pathophysiology of PD with an increased risk of depression.^37^ and maybe BD. The evidence supporting this hypothesis are:

[1] serotonergic activity is reduced in PD;^38^
[2] animal studies have shown that serotonin has the ability to inhibit the release of dopamine from the striatum, where it could be concluded that the reduction in serotonergic activity leads to less inhibition and an increased level of dopamine;^37^ and
[3] reduction inserotonergic tone is a known risk factor for depression. It is likely that many biochemical pathways may cause PD, DA and BD, expressed differently in the beginning and end. The serotonergic hypothesis explains cases where BD is the initial picture.

In conclusion, although serotonin is not clearly involved in the pathophysiology of PD and the serotonergic hypothesis remains controversial, our case report supports the association between PD and BD. The development of mood disorders related to serotonin may be an inadequate adaptation to prevent the emergence of the parkinsonian picture.
